# Bell’s palsy misdiagnosis: characteristics of occult tumors causing facial paralysis

**DOI:** 10.1186/s40463-022-00591-9

**Published:** 2022-10-18

**Authors:** Eun-Jae Chung, Damir Matic, Kevin Fung, S. Danielle MacNeil, Anthony C. Nichols, Ruba Kiwan, KengYeow Tay, John Yoo

**Affiliations:** 1 Department of Otolaryngology-Head and Neck Surgery, London Health Sciences Centre, Schulich School of Medicine and Dentistry, Victoria Hospital, Western University, Room B3-433, 800 Commissioners Road East, London, ON N6A 5W9 Canada; 2 Department of Otorhinolaryngology – Head and Neck Surgery, Seoul National University Hospital, Seoul National University College of Medicine, Seoul, Korea; 3 Division of Plastic and Reconstructive Surgery, Department of Surgery, London Health Sciences Centre, Schulich School of Medicine and Dentistry, Western University, London, ON Canada; 4 Department of Radiology, London Health Sciences Centre, Schulich School of Medicine and Dentistry, Western University, London, ON Canada

**Keywords:** Facial paralysis, Bell’s palsy, Facial nerve, Facial reconstruction, Facial nerve neoplasm

## Abstract

**Objective:**

The aim of this study was to report the incidence and clinical course of a series of patients who were misdiagnosed with Bell’s palsy and were eventually proven to have occult neoplasms.

**Methods:**

Two hundred forty patients with unilateral facial paralysis who were assessed at the facial nerve reanimation clinic, Victoria Hospital, London Health Science Centre, from 2008 through 2017 were reviewed. Persistent paralysis without recovery was the presenting complaint.

**Results:**

Nine patients (3.8%) who were proven to have occult neoplasms initially presented with a diagnosis of Bell’s palsy. The mean diagnostic delay was 43.5 months. Four patients were proven to have skin cancers, 3 patients had parotid cancers, and 2 patients had facial nerve schwannomas as a final diagnosis. Initial magnetic resonance imaging (MRI) was performed in all 9 patients and 8 underwent a follow-up MRI. An occult tumor was identified upon review of the original MRI in one patient and at follow-up MRI in 8 patients. The mean time interval between the initial and follow-up imaging was 30.8 months. The disease status at most recent follow-up were no evidence of disease in 2 patients (22%) and alive with disease in 7 patients (78%). An irreversible, progressive pattern of facial paralysis combined with pain, multiple cranial neuropathies or history of skin cancer were predictable risk factors for occult tumors. Seven out of the 9 patients (77.8%) underwent at least one type of facial reanimation surgery, and the final subjective results by the surgeon were available for 5 patients. Three out of the 5 (60%) patients who were available for final subjective analysis were reported as Grade III according to the modified House-Brackmann scale.

**Conclusion:**

Occult facial nerve neoplasm should be suspected in patients with progressive and irreversible facial paralysis but the diagnosis may only become evident with follow-up imaging. Facial reanimation surgery is a satisfactory option for these patients.

**Graphical abstract:**

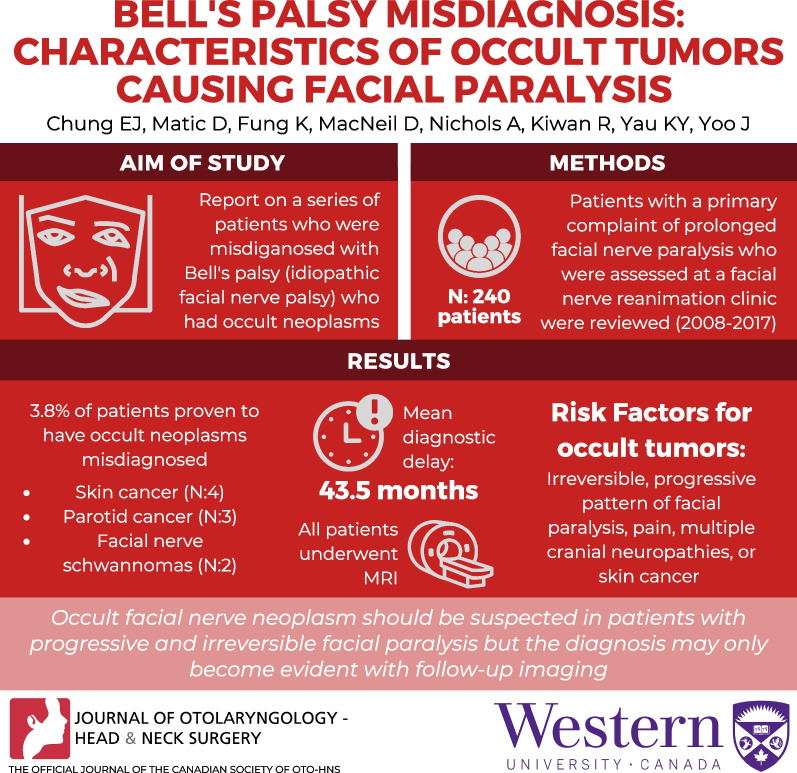

**Supplementary Information:**

The online version contains supplementary material available at 10.1186/s40463-022-00591-9.

## Introduction

Approximately 20% of facial paralysis can be demonstrated to have specific causes, such as infections, inflammatory processes, or neoplasms and facial paralysis can be the first sign of a serious underlying disorder. Eighty percent of all peripheral facial nerve paralysis is initially labeled as idiopathic nerve paralysis, also known as Bell’s palsy. Recovery is usually prompt and complete in 70% of these cases [1]. Neoplasms of the facial nerve is uncommon. It accounts for approximately 5% of all cases [2]. However, there has been no detailed report on the etiology of diagnostic delay, imaging analysis, long-term prognosis and functional results of facial reanimation for these patients. The purpose of this article was to analyze the incidence and clinical course of a series of patients who were misdiagnosed as having Bell’s palsy and were eventually proven to have an occult neoplasm in a single facial nerve reanimation clinic.

## Materials and methods

### Patient demographics

The records of 240 patients with unilateral facial paralysis who were seen at the facial nerve reanimation clinic, Victoria Hospital, London Health Science Centre, from 2008 through 2017 were retrospectively reviewed in the study. The study group was composed of 121 men and 119 women (mean age: 57.3 years; range: 9–95 years).

Prolonged pattern of paralysis without recovery was the main complaint at our facial nerve reanimation clinic. Sixty-three of the 240 patients (26%) were diagnosed as having Bell’s palsy. One hundred of the 240 patients (42%) were diagnosed with facial paralysis associated with tumor (parotid gland in 19 patients, acoustic neuroma in 27 patients, facial nerve schwannoma in 9 patients, skin cancers in 23 patients and other head and neck or skull base tumors in 22 patients). Thirty-eight of the 240 patients were associated with trauma (16%; iatrogenic in 15 and accident or stab wound in 23 patients) and 24 patients (10%) were diagnosed with facial paralysis associated with infection (Ramsay-Hunt syndrome in 19 patients and other infections in 5 patients). Fifteen patients (6%) were identified as having facial paralysis in association with recognizable congenital anomalies. Nine of the 63 patients (14.3%) who were referred with a clinical and MRI diagnosis of Bell’s palsy were proven to have occult neoplasms.

### Diagnostic imaging and outcome analysis

Two experienced head and neck neuroradiologists (R.K. and KY.T.) analyzed all MRIs. The treatment modality of occult neoplasm was determined on the basis of multidisciplinary team approaches, considering several factors, including size and site of the tumor, possibility of curative resection, general performance status of the patient, and preference of each patient. The tumor responses were evaluated using the RECIST 1.1 criteria [3].

### Statistical analysis

The groups were compared using the chi-square to identify differences in presenting symptoms when comparing those with true Bell’s to to those with an occult malignancy. Multivariate analysis was performed by logistic regression using IBM SPSS Statistics version 23.0 (IBM Corp., Armonk, NY, USA). A P value of less than 0.05 was considered significant.

## Results

### Patient demographics

Clinical characteristics, treatment outcomes and pathologic features for the 9 patients who were proven to have occult neoplasms are summarized in Table 1. There were 6 men and 3 women. The mean age was 59.9 years (range, 33–79). Four of the 9 (44.4%) patients were proven to have squamous cell carcinoma (SCC) of the skin, 3 patients (33.3%) had parotid cancer (2 adenoid cystic carcinoma and 1 unknown cell type), and 2 patients (22.3%) had facial nerve schwannomas as the final diagnosis. Patients were referred to the clinic at a mean of 17 months (range 8–28 months, excluding outlier patient 8) after the development of facial paralysis. The mean diagnostic delay (time between initial facial paralysis and diagnosis of occult neoplasm) was 24.5 months (range 15–34 months, excluding outlier patient 8). At the initial consultation at our department, all these patients presented with House-Brackman Grade V or VI facial palsy. Two of the 9 patients (22.2%) developed sudden onset complete facial paralysis. The other 7 patients (77.8%) developed a progressive, irreversible pattern of facial paralysis involving all branches.

**Table 1:** The clinical findings, pathologic features and treatment outcomes of the occult neoplasm of facial nerve

Patient	Sex/age	Final diagnosis	Time to referral (mo)	Initial HB grade	Suspected occult neoplasm	Diagnostic Procedure	Recurrence	Follow-up (mo)	Final outcome
			Diagnostic delay (mo)*	HB grade at referral	Reason for suspicion	Definitive treatment			
1	F/69	Skin SCC	13	2	Yes	Facial nerve biopsy (parotidectomy approach)	No	46	NED
			24	6	Progressive, irreversible	Chemoradiation treatment			
2	M/33	Parotid ACC	8	5	No	Facial nerve biopsy (Incidental finding at facial reanimation surgery)	Regional recurrence, POD 40 mo- > Palliative treatment	68	AWD until last follow-up
			15	5	Irreversible	Radical parotidectomy with neck dissection, temporal bone resection Postoperative radiation			
3	M/58	Skin SCC	20	4	Yes	Transoral biopsy of inferior alveolar nerve (multiple cranial nerve invasion)	Stable disease	22	AWD
			21	5	Progressive, irreversible	RT			
4	M/50	Parotid ACC	15	2	Yes	Facial nerve biopsy	Lung metastasis, POD 32 mo-> salvage surgery	72	NED
			19	6	Progressive, irreversible	Radical parotidectomy with neck dissection, temporal bone resection postoperative radiation			
5	M/54	Skin SCC	12	2	Yes	Infraorbital nerve biopsy	Stable disease	30	AWD
			21	6	Progressive, irreversible	CCRT			
6	M/81	Skin SCC	28	5	Yes	Transmastoid biopsy	Progressive disease	36	AWD until last follow-up
			34	6	irreversible	Clinical trial†			
7	M/79	Facial nerve schwannoma	17	2	Yes	No biopsy‡	Stable disease	28	AWD
			28	6	Progressive, irreversible	Regular follow-up			
8	F/56	Facial nerve schwannoma	180	3	Yes	No biopsy‡	Stable Disease	24	AWD
			180	6	Progressive, irreversible	Regular follow-up			
9	F/59	Parotid malignant tumor	23	3	Yes	Contralateral neck node biopsy		4	AWD until last follow-up
			34	6	Progressive, irreversible	Recommend surgery and postoperative radiation therapy			

*HB* House-Brackmann; *SCC* squamous cell carcinoma; *ACC* adenoid cystic carcinoma; *NED* no evidence of disease; *AWD* alive with disease

^*^Diagnosis delay was defined as the time between initial symptoms of facial weakness and identification of malignancy

^†^Extensive tumor with widespread perineural and dural invasion at the time of pathologic diagnosis

^‡^No surgery for tumor resection of infratemporal facial nerve schwannoma

Occult neoplasm was suspected as the etiology of facial paralysis based on clinical pattern, in 8 patients at the first visit at our clinic, although they were transferred with the initial diagnosis of Bell’s palsy. Facial nerve (n = 4, 44.4%), infraorbital nerve (n = 1, 11.1%), and inferior alveolar nerve (n = 1, 11.1%) biopsies were undertaken as diagnostic procedures. Metastatic parotid malignant tumor was identified with a fine needle aspiration biopsy in one patient. All patients with facial nerve schwannomas were diagnosed with follow-up imaging without facial nerve exploration.

### Diagnostic imaging and outcome analysis

MRI was performed in all 9 patients to determine the etiology before or after the 1^st^ visit to our department. The mean time between initial facial paralysis and the time of 1^st^ imaging was 7.1 months (range 3–14 months). Eight out of the 9 patients underwent follow-up MR at our institute before the identification of occult neoplasm, and the occult tumor was identified at follow-up MR in all 8 patients. The mean time interval between initial and follow-up imaging was 30.8 months (range 2–174 months) (Table 2). The occult tumor was identified at exploration surgery without follow-up MR in the other patient (patient 2).

**Table 2:** Results of the imaging study of occult neoplasm patients

Patient	Imaging study	Time to 1st image	1st image at other hospital	No of imaging before diagnosis	Initial imaging finding	Time interval to follow up image	Interval progression	Reason for diagnostic delay
1 Skin SCC	MR Head	13	no	2	FN enhancement	9	More prominent FN enhancement	Nonspecific FN enhancement
2 Parotid ACC	MR Head	5	yes	1	Normal (report only, other hospital)	N/A*	N/A	Normal in initial MR
3 Skin SCC	MR Head	14	yes	2	Normal	6	Multiple cranial nerve enhancement	Normal in initial MR
4 Parotid ACC	MR Head	5	yes	2	FN enhancement	11	More prominent FN enhancement	Nonspecific FN enhancement
5 Skin SCC	MR Head	3	yes	2	Normal (report only, other hospital)	24	Multiple cranial nerve enhancement	Unknown
6 Skin SCC	MR Head	6	yes	2	Normal (report only, other hospital)	**7**	abnormal enhancement in FN and parotid gland	Unknown
7 Facial nerve schwannoma	MR Head	4	yes	2	Normal (report only, other hospital)	2	Facial nerve focal tumor	Unknown
8 Facial nerve schwannoma	MR Head	6	yes	2	Normal (report only, other hospital)	174	Facial nerve focal tumor	Unknown
9 Parotid malignancy	MR Head	5	yes	3	Normal (report only, other hospital)	12	Parotid tumor with multiple neck metastasis	Misdiagnosis†

*No* number; *FN* facial nerve; *SCC* squamous cell carcinoma; *ACC* adenoid cystic carcinoma

^*^The occult tumor was identified at exploration surgery without follow-up MR

^†^Although the formal radiologic reports of the initial MR image from the radiologist at the outside hospital were compatible with Bell’s palsy, our neuroradiologist re-evaluated the initial MR image and found a small suspicious lesion in the parotid deep lobe

**Fig. 1:**
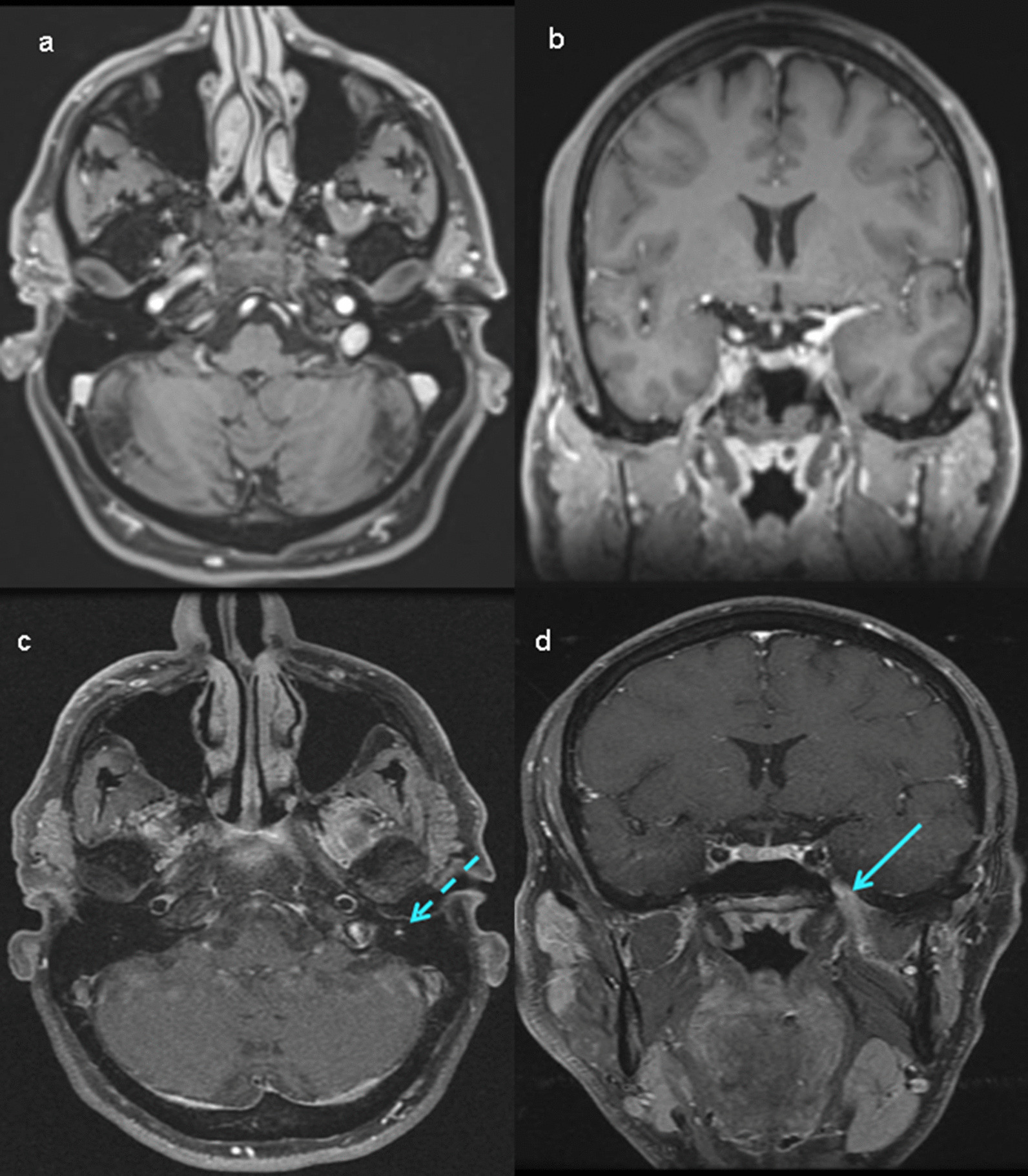
Comparison between initial and follow-T1 Post-gadolinium MR imaging in patient 3 **a** axial view **b** coronal view. No abnormality identified. MRI 6 months later **c** axial image shows abnormal asymmetric enhancement in the mastoid segment of left facial nerve (dashed arrow) **d** coronal image shows extension along the V3 branch of the left trigeminal nerve (arrow)

Initial MR imaging was normal in one out of 9 patients (11.1%, patient 3). Abnormal asymmetric enhancement in the mastoid segment of the facial nerve developed at the follow-up MR image at 6 months, and the lesion was identified along the V3 branch of the trigeminal nerve in this patient (Fig. 1). The result of transoral biopsy of the inferior alveolar nerve was squamous cell carcinoma. The patient was in a state of stable disease (SD) after definitive radiotherapy (RT) at 22 months following treatment.

**Fig. 2:**
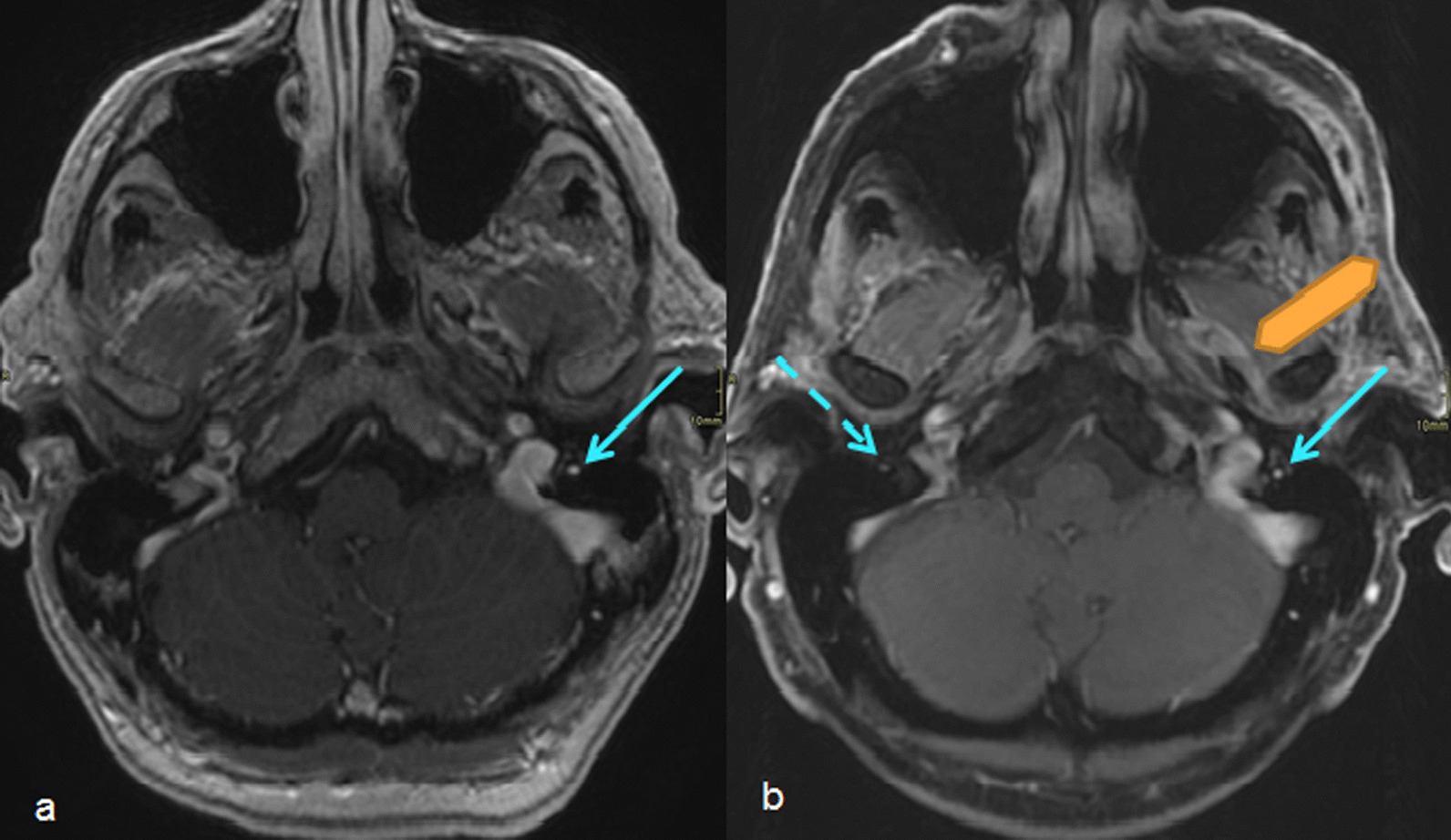
Comparison between initial and follow-up axial T1 Post-gadolinium MR imaging in patient 1 **a** MRI shows asymmetric enhancement of the mastoid segment of the left CN VII (arrow) **b** Follow-up MRI 12 months later shows asymmetric enhancement in the left masticator space extending medially along the expected course of the auriculotemporal nerve (double arrow), a branch of the left trigeminal nerve

**Fig. 3:**
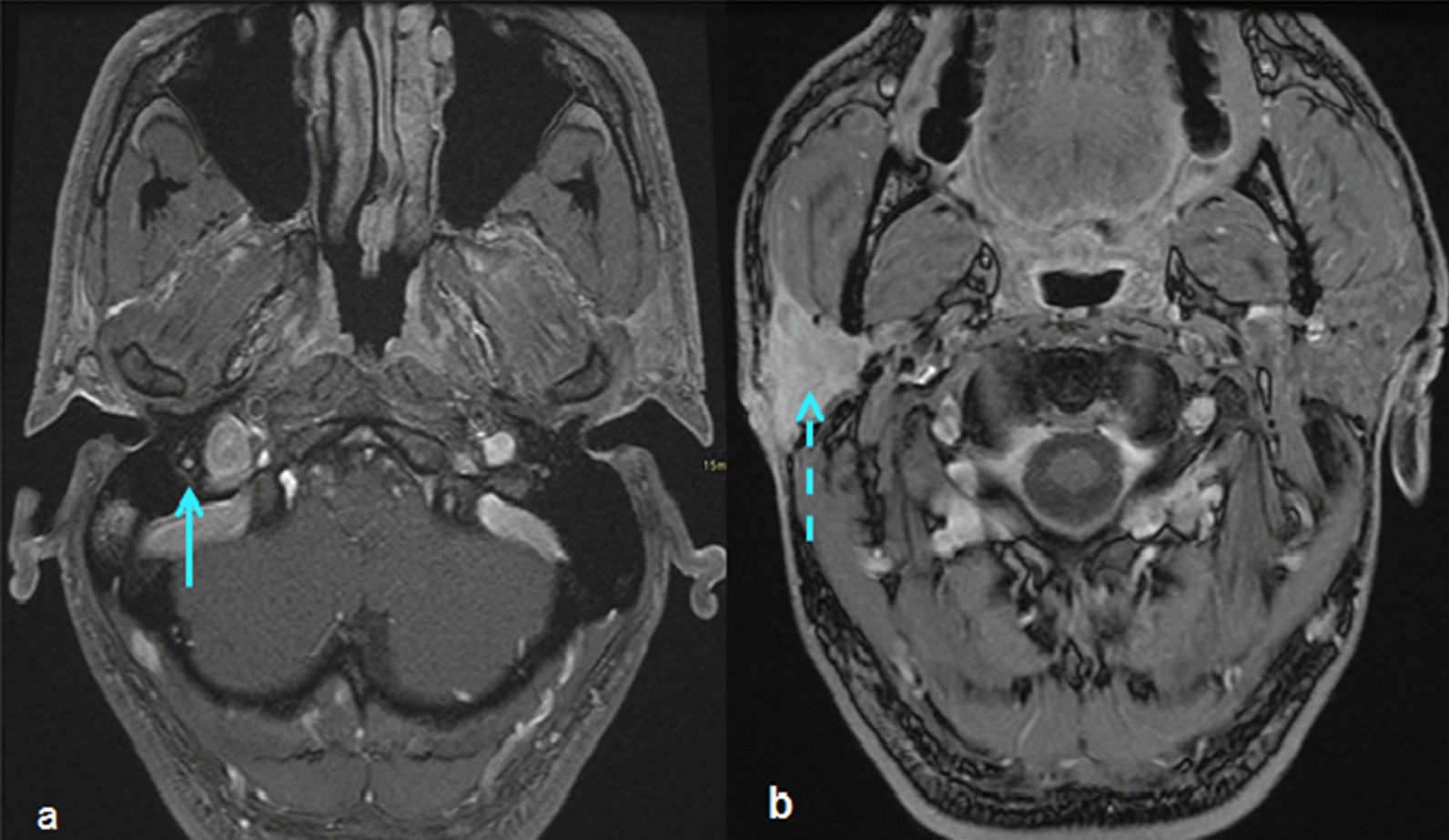
Comparison between initial and follow-up axial T1 Post- gadolinium MR imaging in patient 4 **a** asymmetric enhancement at the mastoid segment of right facial nerve (arrow) **b** MRI 3 weeks later with full coverage of the parotid gland shows obvious enhancement of the right parotid gland (dashed arrow)

Nonspecific enhancement of the facial nerve was reported in 2 patients (22.2%) at the initial MR. The extent of asymmetric enhancement was increased in these two patients at the follow-up MRI (Figs. 2 and 3). The final pathologic diagnoses were squamous cell carcinoma of the skin (patient 1) and adenoid cystic carcinoma of parotid gland (patient 4). Primary chemoradiation treatment was recommended and complete response (CR) was seen in patient 1. Patient 4 underwent radical surgery and postoperative radiation treatment. The patient developed lung metastasis, and subsequently underwent salvage surgery for isolated lung metastasis with video-assisted thoracoscopic surgery. Patients 1 and 4 were alive without significant morbidity at the time of this report.

In 3 patients (patient 3, 4 and 9), the initial MRI conducted at an outside hospital prior to referral were re-evaluated by our neuroradiologists following their initial clinic visit. In one case, a small suspicious lesion in the parotid deep lobe was found (patient 9). This patient was lost to follow-up. In 5 cases, only the dictated MRI reports (consistent with Bell’s palsy) from the outside hospital without images were available (patient 2, 5, 6, 7 and 8) and repeat imaging was performed at our institution. The final diagnosis of these patients were facial nerve schwannoma in 2 patients (patients 7 and 8), skin squamous cell carcinoma in 2 patients (patient 5 and 6) and parotid adenoid cystic carcinoma in 1 patient (patient 2). All patients with facial nerve schwannoma refused surgical resection of infratemporal tumor. Of the two patients with skin cancer, one patient (patient 5) underwent definitive chemoradiotherapy (CRT) and stable disease (SD) was achieved. A clinical trial was recommended for the other patient with skin cancer because of extensive perineural and dural invasion (patient 6). Radical surgery and postoperative radiation treatment was performed for the other parotid cancer patient who underwent surgical exploration without follow-up MR (patient No. 2). This patient developed regional recurrence that was not considered for salvage treatment because of the extensive perineural extension.

The final outcomes of the occult tumor patients (n = 9) were no evidence of disease in 2 patients (22%) and alive with disease in 7 patients (78%) during a mean follow-up of 36.7 months (range 4–72 months).

### Risk factor analysis of occult neoplasm

To determine the risk factors for occult neoplasms of the facial nerve, we compared various clinical factors in patients with true Bell’s palsy (Table 3). Among the 63 patients with Bell’s palsy in our database, 32 patients with inadequate information were excluded. All patients in the occult neoplasm group presented with complete and irreversible facial paralysis of greater than 6 months duration (*p* < 0.001). A history of progressive facial nerve paralysis with sequential involvement of adjacent branches was typical in most patients. Seven of the nine (77.8%) patients in the occult neoplasm group developed a progressive pattern of paralysis and 4 out of the 9 (44.4%) patients developed hypesthesia. Facial or retroauricular pain was another associated characteristic in 6 out of the 9 (66.7%) patients. Three patients (33.3%) had a history of regional skin squamous cell carcinoma.

**Table 3:** The risk factor analysis of occult neoplasm

	Bell’s palsy	Occult neoplasm	*p* value
Male	14/32 (43.8%)	6/9 (66.7%)	0.277
Age ≥ 65	9/32 (28.1%)	3/9 (33.3%)	0.762
Facial or retroauricular pain	7/32 (21.9%)	6/9 (66.7%)	0.011*
Hypesthesia or dysesthesia	3/32 (9.4%)	4/9 (44.4%)	0.014*
Dysgeusia	3/32 (9.4%)	0/9 (0%)	0.818
Facial twitching	1/32 (3.1%)	1/9 (11.1%)	0.370
Sudden onset (< 72 h)	31/32 (96.9%)	2/9 (22.2%)	< 0.001*
Progressive	1/32 (3.1%)	7/9 (77.8%)	< 0.001*
Recurrent	3/32 (9.4%)	1/9 (11.1%)	0.878
Complete palsy (> 6 months)	1/32 (3.1%)	9/9 (100%)	< 0.001*
Irreversible, no improvement (> 6 months)	0/32 (0%)	9/9 (100%)	< 0.001*
History of skin cancer	1/32 (3.1%)	3/9 (33.3%)	0.07*
Total	32*	9	

*Among the 63 patients with Bell’s palsy in our database, 32 patients with inadequate information were excluded

### Facial reanimation surgery

Seven out of 9 the patients (77.8%) underwent at least one type of facial reanimation surgery. Three patients underwent facial reanimation as a concurrent operation with ablation surgery, and 4 patients had a staged operation. The mean number of reanimation surgeries was 1.6 (range 0–3). Surgery for the upper face included upper eyelid gold weight (n = 6, 85.7%) and lower lid tendon suspension (n = 3, 42.8%) and nerve interposition cable graft (n = 1, 14.3%). Static suspension to midface and mouth (n = 4, 57.1%), dynamic suspension (n = 2, 28.6%). and gracilis free flap (n = 2, 28.6%) were used for the lower face reanimations.

**Fig. 4:**
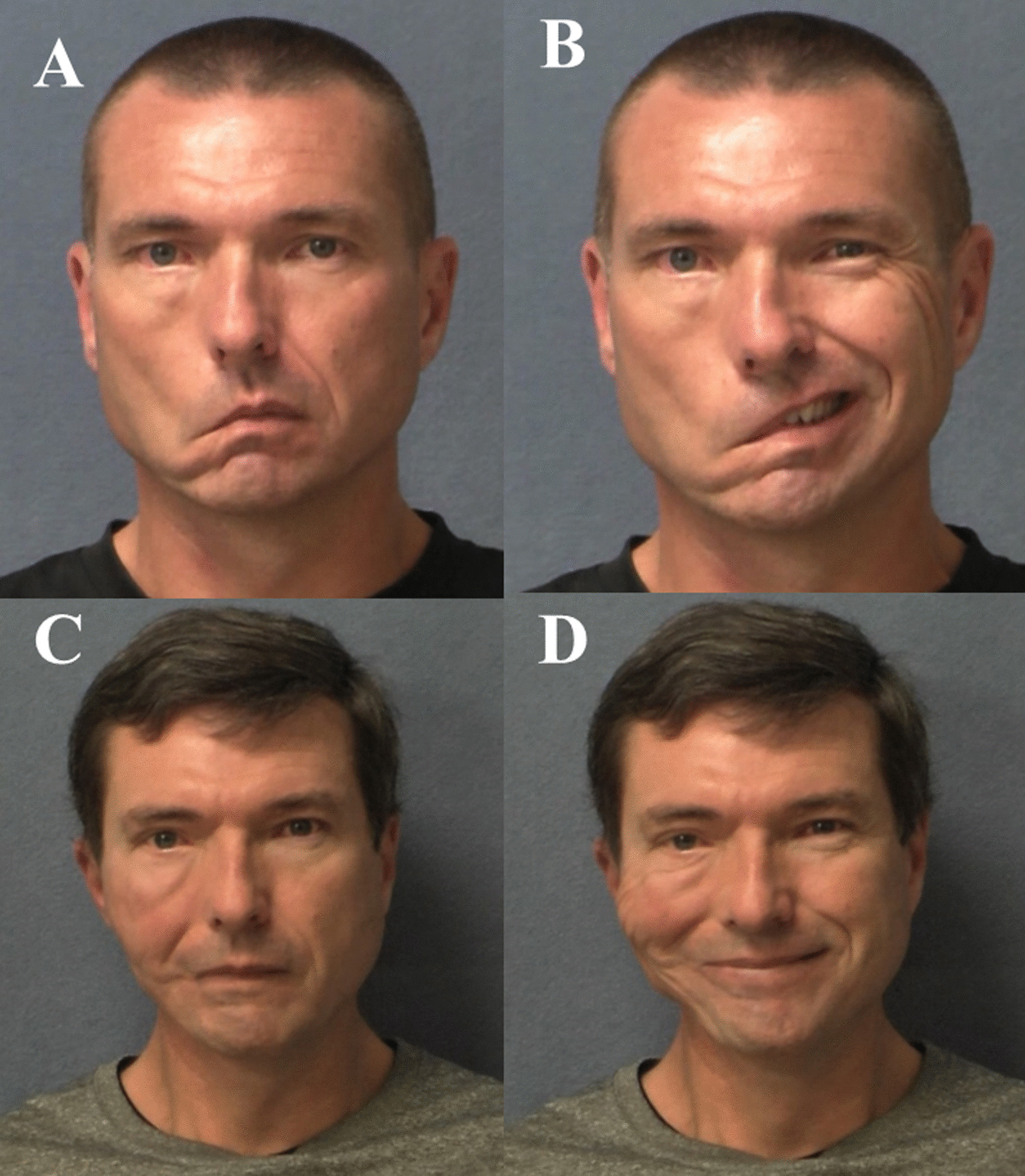
Final outcome after facial reanimation surgery in patient 4. Gold weight implant, lower lid tendon suspension, and gracilis **a** preoperative resting **b** preoperative smile **c** 13 months postoperative with resting **d** 13 months postoperative with smile

The postoperative video files of these patients (postoperative 6–12 months) were evaluated retrospectively by at least 2 physicians, including the primary surgeon (J.Y.) according to the modified House-Brackmann scale, as suggested by the Sir Charles Bell Society proposal (Additional file 1: Table S1) [4, 5]. The final subjective results by the surgeon at the final follow-up were available for 5 patients (Table 4). Three out of the 5 (60%) patients were reported as having grade III, and the other 2 patients (40%) were reported as having grades IV and V. All patients with grade IV or V underwent (C)RT as their definite treatment for the neurotropic cutaneous squamous cell carcinoma (Fig. 4 and Additional file 2: video S1).

**Table 4:** Facial reanimation surgery

Patient	Reanimation surgery	No of reanimation surgeries	Upper face	Lower face	Final result at last follow-up (modified House-Brackmann Grade)						
					Eyebrow	Eye	Nasolabial fold	Oral	Whole face	Synkinesis	Grade
1	Concurrent	2	Gold weight insertion Brow lift Lower lid sling	Static sling Lower lip wedge resection	4	2	4	4	3	1	IV
2	Concurrent	1	Facial nerve interposition cable graft	Gracilis free tissue transfer Additional dynamic suspension using temporalis muscle	3	3	1	1	2	0	III
3	Stage	1	Gold weight insertion	Static sling	4	4	4	4	4	0	V
4	Concurrent	3	Gold weight insertion, Lower lid sling	Gracilis free tissue transfer	4	2	1	1	2	0	III
5	Stage	2	Gold weight insertion	Orthodromic temporalis muscle transfer	4	1	3	3	3	0	III
6	None	0	Refuse	Refuse	N/A	N/A	N/A	N/A	N/A	N/A	N/A
7	Stage	1	Gold weight insertion, Lower lid sling*	Static sling*	N/A	N/A	N/A	N/A	N/A	N/A	N/A
8	None	0	Refuse	Refuse	N/A	N/A	N/A	N/A	N/A	N/A	N/A
9	Stage	1	Gold weight insertion *	Static sling*	N/A	N/A	N/A	N/A	N/A	N/A	N/A

*Surgery was performed at another hospital

## Discussion

Many etiological factors can cause unilateral facial palsy but Bell’s palsy, is the most common diagnosis. In the report of 1989 patients presenting to a tertiary facial nerve center, Bell’s palsy accounted for 38%, acoustic neuroma resections 10%, cancer 7%, iatrogenic injuries 7%, varicella zoster 7%, benign lesions 5%, congenital palsy 5%, Lyme disease 4%, and other causes 17%. [6] There has been no report on the analysis of etiologies from a facial nerve reanimation clinic that mainly offers interventions to help restore a more normal appearance. The prolonged pattern of paralysis without recovery was the main complaint at our facial nerve reanimation clinic. Forty-two percent of the patients were diagnosed with facial paralysis associated with tumors, 26% with Bell’s palsy, 16% with trauma, 10% with infection and 6% with congenital anomalies. There was a significantly higher rate of tumors compared to results of previous reports [6].

Bell’s palsy is the most common cause of facial nerve paralysis or weakness and is typically self-limited. Bell’s palsy may be associated with mild pain, numbness, increased sensitivity to sound, and alterations in taste. Onset is usually rapid (< 72 h), with spontaneous recovery occurring within 2 to 8 weeks in more than 80% of patients. Although Bell palsy has a classic presentation readily identified with a thorough history and physical exam, it remains a diagnosis of exclusion after other potential causes are ruled out [7].

Facial paralysis of neoplastic origin is estimated to represent the etiology in approximately 5% of all cases [2]. Neoplastic origin may be neurogenic primary lesions of the seventh cranial nerve or secondary, extrinsic neoplasms [2]. We analyzed 9 patients with unilateral facial paralysis, all of whom had an initial diagnosis of Bell’s palsy and were ultimately diagnosed with occult neoplasms. The formal radiologic report of the initial MRI of the patients reported no evidence of tumorous lesions. We doubted the diagnoses of Bell’s palsy based on the unusual histories that were inconsistent with Bell’s palsy with respect to the progressive and irreversible unilateral facial paralysis in all patients. Persistent facial paralysis with no return of any function beyond 6 months is usually not idiopathic [8].

The diagnosis of Bell palsy based on the MRI findings in some situations may lead to a false sense of security for patients with unilateral facial paralysis. Despite the dramatic advances in technologies of the various diagnostic modalities, Jackson et al. wrote that there remain limitations of resolution, and false-negative results are not uncommon [9]. Surgical exploration of the facial nerve may serves as a diagnostic and therapeutic approach in selected circumstances [9]. Surgical exploration of the parotid gland and facial nerve should be considered in patients with facial nerve paralysis who show no signs of regeneration 6 months after the onset of paralysis and/or persistent electrophysiological signs of ongoing neuronal degeneration, even if imaging tests, such as MRI studies, show no evidence of tumor [2].

If the facial nerve paralysis progresses slowly to complete paralysis and persists without any sign of recovery, it strongly suggests a neoplastic lesion involving the facial nerve. A slow progression of facial paralysis is the clinical characteristic of the neoplastic disorder, in contrast with the typical brief and self-limited course of Bell palsy. Nevertheless, a sudden onset of facial paralysis does not always exclude tumor involvement of the facial nerve. Progressing unilateral facial paralysis beyond 3 weeks and no evidence of recovery after 6 months of paralysis strongly suggest neoplastic involvement in the facial nerve [1].

Pain was also the main associated complaint in our cohort (6 out of the 9 patients; 66.7%). Although facial pain is not unusual in patients with Ramsey-Hunt syndrome or Bell’s palsy, its presence must be investigated. Jackson and Glasscock [9] reported that pain was identified in 18% of their patients with facial nerve neoplasm. Persistent pain beyond several months after onset of facial paralysis should warrant further investigation for an occult neoplasm [8].

A cranial neuropathy other than the facial nerve is sometimes seen in patients with Bell palsy. However, a high level of suspicion is necessary when there are persistent sensory and motor deficits beyond the facial nerve [8] .

Previous treatment history of regional skin cancer is also known to be a strong causal factor for facial nerve paralysis, especially in patients with pain involving other nerves [8]. In our series, 3 of the 9 patients had a history of a regional skin squamous cell carcinoma.

In our cohort, the possible reasons for diagnostic delay were no abnormal findings on the initial image, nonspecific facial nerve enhancement on initial MR, or non-enhancement with gadolinium. Therefore, follow-up imaging with adequate coverage of the entire facial nerve and neuroradiology consultation appears to be an essential component to mitigate diagnostic delay. In our, patient 2 did not receive repeat imaging prior to surgery as no occult neoplasm was suspected. This patient was a 33-year-old-male with a typical history of right Bell's palsy. Quality and severity did not change over 8 months. At that time, there were only MRI report from other center (without consultation from neuroradiologist) and the report of MRI did not show any etiology. Four months later, he underwent reconstructive surgery after extensive consultation. The facial nerve was identified through a retrograde approach, and we found completely abnormal and infiltrative tumor invading the facial nerve. It was small but very hard mass arising from deep to the facial nerve extending into the parapharyngeal space. The final diagnosis was high-grade adenoid cystic carcinoma measuring at least 2 cm in size, with perineural infiltration and microscopically positive margin. After surgery, he received adjuvant radiation treatment. This would be an example of the occult neoplasm identified by consulting a neuroradiologist or repeating MRI images.

None of the nine patients would have been confirmed to have a neoplasm without rigorous and long-term follow up. Therefore, careful and deliberate follow-up incorporating appropriate imaging evaluated by expert radiologists is essential when confronted with atypical presentations of facial paralysis.

The approach to re-animation is especially challenging for these patients because of the diagnostic dilemma coupled with limited options due to their late presentations. In our study, seven out of the 9 patients (77.8%) underwent at least one type of facial reanimation surgery. Three out of the five (60%) patients were graded as HB III with satisfactory symmetry, mouth angle excursion and eye protection. Even in patients with occult neoplasms and those alive with disease, facial reanimation surgery an improve quality of life in selected patients.

The limitations of this study were that because of the low number of patients with occult neoplasms making strong recommendations  should be done with caution. The source of initial HB grade may not be accurate because of the retrospective nature of this study.

## Conclusion

Occult facial nerve neoplasm should be suspected in patients with progressive and irreversible facial paralysis. Repeat MRI and surgical exploration may be required to make the diagnosis. Facial reanimation surgery should be offered for these patients.

## Supplementary Information

**Additional file 1**. ** Table S1**. Modified House-Brackmann grading sale system.

**Additional file 2**.** Table S2**. Preop and postop videos of patient 4 and corresponds to Fig. 4. Gold weight implant, lower lid tendon suspension, and gracilis.

## Data Availability

The datasets used and analysed during the current study are available from the corresponding author on reasonable request.
