# Supplementary material for: Bell’s palsy misdiagnosis: characteristics of occult tumors causing facial paralysis
Source: J Otolaryngol Head Neck Surg. 2022 Oct 18;51:39. doi: 10.1186/s40463-022-00591-9 (PMC9580210; doi:10.1186/s40463-022-00591-9)
Supplement: Supplementary file 1 — Additional file 1. Table S1. Modified House-Brackmann grading sale system. [file 40463_2022_591_MOESM1_ESM.docx]

**Supplementary Table 1.** Modified House-Brackmann grading sale system

| Facial Zone | Movement (%) | Movement Score | Synkinesis (quantity) | Synkinesis Score | Total Score | Grade |
| --- | --- | --- | --- | --- | --- | --- |
| Eyebrow | 100 | 1 | None | 0 | 4 | I |
| Eye | >75 | 2 | Slight | 1 | 5–9 | II |
| Nasolabial fold | >50 | 3 | Obvious | 2 | 10–14 | III |
| Oral | <50 | 4 | Disfiguring | 3 | 15–19 | IV |
|  | Poor | 5 |  |  | 20–23 | V |
| Whole face | None | 6 |  |  | 24 | VI |

* Four facial zones (eyebrow, eye, nasolabial fold, and oral) are graded according to percentages of movement and quantity of synkinesis. The

sum of scores leads to grade (Roman numeral).
